# Gas exchange characteristics and their influencing factors for halophytic plant communities on west coast of Bohai Sea

**DOI:** 10.1371/journal.pone.0229047

**Published:** 2020-02-12

**Authors:** Fude Liu, Xue Mo, Sen Zhang, Feijie Chen, Desheng Li

**Affiliations:** School of Environmental Science and Safety Engineering, Tianjin University of Technology, Tianjin, P. R. China; Shandong University, CHINA

## Abstract

Water–salt stress and nutrient limitation may affect leaf economic spectrum of halophytes and confuse our understanding on plant physiological principles in a changing world. In this study, three halophytic plant communities of *Phragmites australis*, *Suaeda salsa*, and *Tamarix chinensis*, were selected in two sites (sites 1 and 2) on the west coast of Bohai Sea. The net photosynthetic rate (*P*_n_), transpiration rate (*T*_r_), stomatal conductance (*G*_s_), leaf vapor pressure deficit (VPD_leaf_) and their influencing factors were studied to test the possible carbon assimilation strategies of the halophytes. *P*. *australis* had higher *P*_n_, *T*_r_, and *G*_s_ than *S*. *salsa* and *T*. *chinensis* in both sites. Similar trends were found for leaf P and photosynthetic N and P efficiency (PNUE and PPUE, respectively) in one or both sites. By contrast, the leaf dry mass per area (LMA) increased in the order of *P*. *australis* < *S*. *salsa* < *T*. *chinensis* in both sites. For identical species in different sites, *P*_n_, leaf P, and PNUE were lower but *T*_r_, VPD_leaf_, leaf N, leaf N:P, and PPUE were higher in site 1 than in site 2 for one or more halophytes. Although soil physicochemical properties in different sites explained several variations among the halophytes, two-way ANOVA indicated that the species can explain most of the leaf traits compared with the site. LMA also had significant nonlinear relationships with *P*_n_, *T*_r_, *G*_s_, and VPD_leaf_. PNUE and PPUE showed positive correlation with *P*_n_ in both sites, but they decreased in the power-law function with increasing LMA. Overall, the redundancy analysis showed that the gas exchange capacity of the halophytic plant communities was significantly affected by PPUE (60.0% of explanation), PNUE (57.1%), LMA (35.0%), leaf P (22.0%), and soil N (15.8%).

## Introduction

Photosynthesis is one of the most important physiological processes in plants [1]. The photosynthetic products, i.e., carbon hydrates, are the basic material and energy source for all living beings [2]. Leaf N and P concentrations are crucial parameters for gas exchange activities [3–5]. Over the past decades, ecologists have become increasingly concerned with quantifying the correlations between photosynthetic characters and leaf nutrients. These ecologists have discovered that foliar N and/or P are positively correlated with the photosynthetic rate (*P*_n_) in regional and global scales [6–10].

The physiological responses of plants to N availability are well-documented [11–13]. For example, the correlations between photosynthetic capacity and leaf N concentration are always shown positively for many plants in terrestrial ecosystems [6–10, 14], because most of the leaf N is invested in photosynthetic apparatus [15, 16]. Although the relationships between photosynthetic capacity and leaf P concentration are not familiar with photosynthesis-leaf N [17, 18], leaf P is correlated with photosynthetic capacity positively under P-limited conditions [4, 9, 19]. However, those correlations are poor under P-rich conditions [20, 21]. Other leaf traits, such as leaf texture and structure, can also affect leaf photosynthesis under many conditions. For example, high leaf dry mass per area (LMA) in a barren environment indicates a large carbohydrate allocation pattern in leaves, thereby leading to a nutrient dilution effect in the photosynthetic apparatus and reducing leaves’ carbon assimilation capacity [22]. The LMA is also strongly correlated with leaf P than with leaf N in P-limited ecosystems [23].

The large-scale quantification for leaf trait relationships has considerably improved our understanding of the leaf economic spectrum, but this spectrum tends to be regulated by soil nutrient supply [24]. Photosynthesis is an environmentally sensitive activity in the changing world [25]. Thus, nutrient deficiency in soils might trigger a series of adjustments, which will eventually lead to fluctuations in the leaf economic spectrum. At present, the world is undergoing a series of climate changes, such as the increase in atmospheric CO_2_, global warming, and N deposition. These phenomena are mainly caused by anthropogenic activities and predicted to increase in the near future [26]. Under these climate change conditions, the primary production of plants is predicted to depend on P but not N availability [27], because the P-limited phenomenon may be more prevalent than we think in the changing world [28]. Although various useful information on P-limited ecosystems such as tropical forests, rivers, and lakes in many areas [9, 29, 30] has been documented, evidence regarding the halophytic plant communities of coastal wetlands is limited.

In general, N is the predominant limiting nutrient of plants in coastal wetland [31]. However, in recent decades, atmospheric N deposition has become widespread in coastal areas due to the increasing population density [32]. The atmospheric P deposition is almost negligible [33], but it results in a high anthropogenic N deposition and high deposited N:P ratios in those coastal regions. The amounts of fertilizer inputs also have increased exponentially since the 1950s, which indicates that coastal waters receive more N than P fertilizers through surface runoff and sea-going rivers [32, 34]. Therefore, the imbalance in N and P inputs leads to a shift from N-limited to P-limited environments in coastal wetlands. This nutrient limitation shift may affect the relationships between leaf traits and ultimately change the leaf economic spectrum of plant. The leaves of halophytes are different from those of other terrestrial plants. Halophytes often have large photosynthetic cells and chloroplast ultrastructures due to their long-term adaptation to salt stress, thereby possibly affecting their leaf economic spectrum and confusing our understanding of plant physiological principles under climate changes.

In this study, three typical halophytic plant communities of *Phragmites australis*, *Suaeda salsa*, and *Tamarix chinensis* were selected in two sites (sites 1 and 2) on the west coast of Bohai Sea. The objectives of this study are as follows: (1) to document the gas exchange characteristics and their correlations with other leaf traits of halophytes in the coastal wetlands; (2) to test the influencing factors of gas exchange capacity of halophytes in the coastal wetlands in nutrient limitation conditions; and (3) to discuss the possible strategies adopted by halophytes in photosynthetic carbon assimilation under the nutrient limitation conditions.

## Materials and methods

### Ethics statement

Our experiment was performed in the Yellow River Delta Nature Reserve and the Beidagang Wetland Nature Reserve, China. This study was approved by the Yellow River Delta Nature Reserve Management Bureau of Shandong and Beidagang Wetland Nature Reserve Management Center of Tianjin. We confirmed that our study has no harm to the environment, and the field studies did not involve endangered or protected species.

### Site description

This study was carried out in two typical coastal wetlands on the west coast of Bohai Sea. Site 1 is located at the Yellow River Delta Nature Reserve (37°35′–38°12′N, 118°33′–119°20′E) of Dongying, China. Dongying is an agricultural high-tech industry demonstration area in Shandong Province. The total area of Yellow River Delta Nature Reserve is 1530 km^2^, with a supratidal area of 827 km^2^, intertidal area of 382.5 km^2^, and shallow sea area (<3 m in low-tide) of 320.5 km^2^. In this study, the halophytic plant communities were selected from coastal wetlands of the supratidal area in site 1. This region has a typical continental monsoon climate. The mean annual temperature, evaporation, and precipitation are 12.3°C, 1926.1 mm, and 542.3 mm, respectively. The frost-free period is 199 days, and the rainy season starts in June and ends in August. Intrazonal tidal soil and salty soil are distributed in this region, and the dominant species are *P*. *australis*, *T*. *chinensis*, and *S*. *salsa* [35]. Site 2 is located on the Beidagang Wetland Nature Reserve (38°36′–38°57′N, 117°11′–117°37′E) of Tianjin, China. In contrast to the agricultural city of Dongying, Tianjin is an important industrial city of China. The total area of Beidagang Wetland Nature Reserve is 348.87 km^2^, with a core area of 115.72 km^2^, buffer area of 91.96 km^2^, and experimental area of 141.19 km^2^. Beidagang Wetland Nature Reserve is the largest wetland nature reserve of Tianjin, and the types of wetlands include coastal wetlands, rivers, swamps, ditches, and other artificial wetlands. The halophytic plant communities were selected from coastal wetlands of the experimental area in site 2. The climate of this region is dominated by a northern subtropical monsoon. The mean annual temperature, evaporation, and precipitation are 11.3°C, 1500 mm, and 600 mm, respectively. The rainy season extends from June to September [36]. The soil types of Tianjin coastal wetlands are meadow solonchak and salinized meadow soil, and the dominant species are herbaceous plants, that is, *S*. *salsa* and *P*. *australis* and shrubs, such as *T*. *chinensis*.

### Photosynthesis measurements

In the wet season of 2015, the three typical halophyte plant communities of *P*. *australis*, *S*. *salsa* and *T*. *chinensis* were chosen at each site. Six quadrats (1 m × 1 m for *P*. *australis* and *S*. *salsa* communities and 3 m × 3 m for *T*. *chinensis* community) were randomly set in each community. In each community, five individuals were selected for photosynthesis measurement. In this research, a portable leaf chamber and open system infrared gas analyzer (CI-340; CID, Inc., USA) were used to measure the photosynthetic characters, including the net photosynthetic rate (*P*_n_), transpiration rate (*T*_r_), stomatal conductance (*G*_s_), and leaf vapor pressure deficit (VPD_leaf_). Similar and uniform sunlit days were selected to minimize the sources of diurnal heterogeneity. The gas analyzer was calibrated daily and checked periodically throughout the measurement period. Measurement was performed from mid-morning to late morning (08:30–10:30, depending on local weather). Leaves with no signs of injury or disease for each individual were measured at ambient CO_2_ concentrations (approximately 370 μmol·mol^−1^) and under natural light conditions. The photosynthetic photon flux density ranged from 1000 μmol·m^−2^·s^−1^ to 1200 μmol·m^−2^·s^−1^, which proved to be saturated for photosynthesis rate. One leaf chamber (CI-301LC-2, CID, Inc., USA) was used for *P*. *australis* measurement. Another leaf chamber (CI-301LC-5, CID, Inc., USA) was used for *S*. *salsa* and *T*. *chinensis* measurement. The air flow rate was 0.3 L·min^-1^, and the ambient temperature ranged from 26°C to 28°C.

### Field sampling

After photosynthesis measurement, the same leaves were removed from the branches of each individual for leaf area measurement. For all halophytes, multiple leaves were collected, and the leaf area was measured by a laser area meter (CI-202, CID, Inc., USA). Then, the leaves were dried in an oven for 65°C for at least 48 h and weighed. LMA was calculated as leaf dry mass divided by leaf area. Finally, all dried leaves were ground into powder for N and P measurements. Five soil samples were also collected randomly at the depth of 0–10 cm in each quadrat. Then, the samples of the same layers were mixed as one sample, placed inside polyethylene bags, and brought to the laboratory. A total of 36 soil samples were collected from both the two sites. All soil samples were air dried and ground into powder for chemical analysis.

### Measurements for plant and soil nutrients

The total N concentrations of plant and soil samples was determined using an elemental analyzer (Vario EL III, Elementar, Germany), whereas the total P concentrations of the plant and soil samples were measured by inductively coupled plasma optical emission spectrometry (Varian, Inc., USA).

### Photosynthetic nutrient use efficiency calculation

Photosynthetic data and leaf N or P were used to calculate the photosynthetic N (PNUE) and P use efficiency (PPUE), as defined by Field and Mooney [14]. PNUE was calculated as the ratio between photosynthetic rate per mass (*P*_n_/LMA) and leaf N concentration, and PPUE was computed as the ratio between photosynthetic rate per mass and leaf P concentration.

### Soil physicochemical property measurements

Soil pH was measured by a potentiometric method with a soil–water ratio of 1:2.5 (IQ-150 Spectrum Technologies, Inc., Germany). The soil water content was measured by a soil moisture measuring instrument (HD2, IMKO, Germany). The soil salt concentration was determined using the weighing method with a soil–water ratio of 1:5. To avoid experimental errors, we repeated all measurements thrice.

### Data analysis

All data used in this study were the arithmetical averages of multiple repetitions (S1 Dataset). Then, one-way ANOVA was used to test the soil physicochemical properties (soil N, soil P, salinity, SWC, and pH), gas exchange parameters (*P*_n_, *T*_r_, *G*_s_, and VPD_leaf_), leaf chemical and structural traits (leaf N, leaf P, leaf N:P, and LMA), and photosynthetic nutrient use efficiency (PNUE and PPUE) of the three halophytic plant communities at the same site. In multiple comparison tests, the Games–Howell method was used when variances were assumed to be heterogeneous using the Levene’s test. Tukey’s method was applied when variances were homogeneous. For identical species between the two sites, the differences were tested by *t*-test. We also used the regression approach to two-way ANOVA as implemented in a general linear model using type Ш sums of squares. The model included species, site, and their interaction (species × site). The significance of the effects was tested using *F*-ratios between the mean squares of effects and residuals. All statistical analyses were conducted in IBM SPSS Statistics 22.0 for Windows (SPSS Inc., Chicago, IL, USA).

A redundancy analysis (RDA) and Monte Carlo test were performed using Canoco 4.5 software to evaluate the relationships between gas exchange characteristics and their influencing factors of all halophytic plant communities, including *P*. *australis*, *S*. *salsa*, and *T*. *chinensis* in sites 1 and 2. A standardized major axis regression was used to describe the pairwise relationships of PNUE-*P*_n_ and PPUE-*P*_n_. The allometric equation parameters were calculated using (S) MATR version 2.0 [37]. The heterogeneity of the regression slopes was tested using a method introduced by Warton and Weber [38]. When the homogeneity of the slope occurred, the differences in the y-intercept of the regression slopes were also tested by WALD test. In the present study, the carbon assimilation strategies of the halophytes in different sites were explained by slopes and y-intercepts.

## Results

### Gas exchange characteristics of halophytes

**Fig 1:**
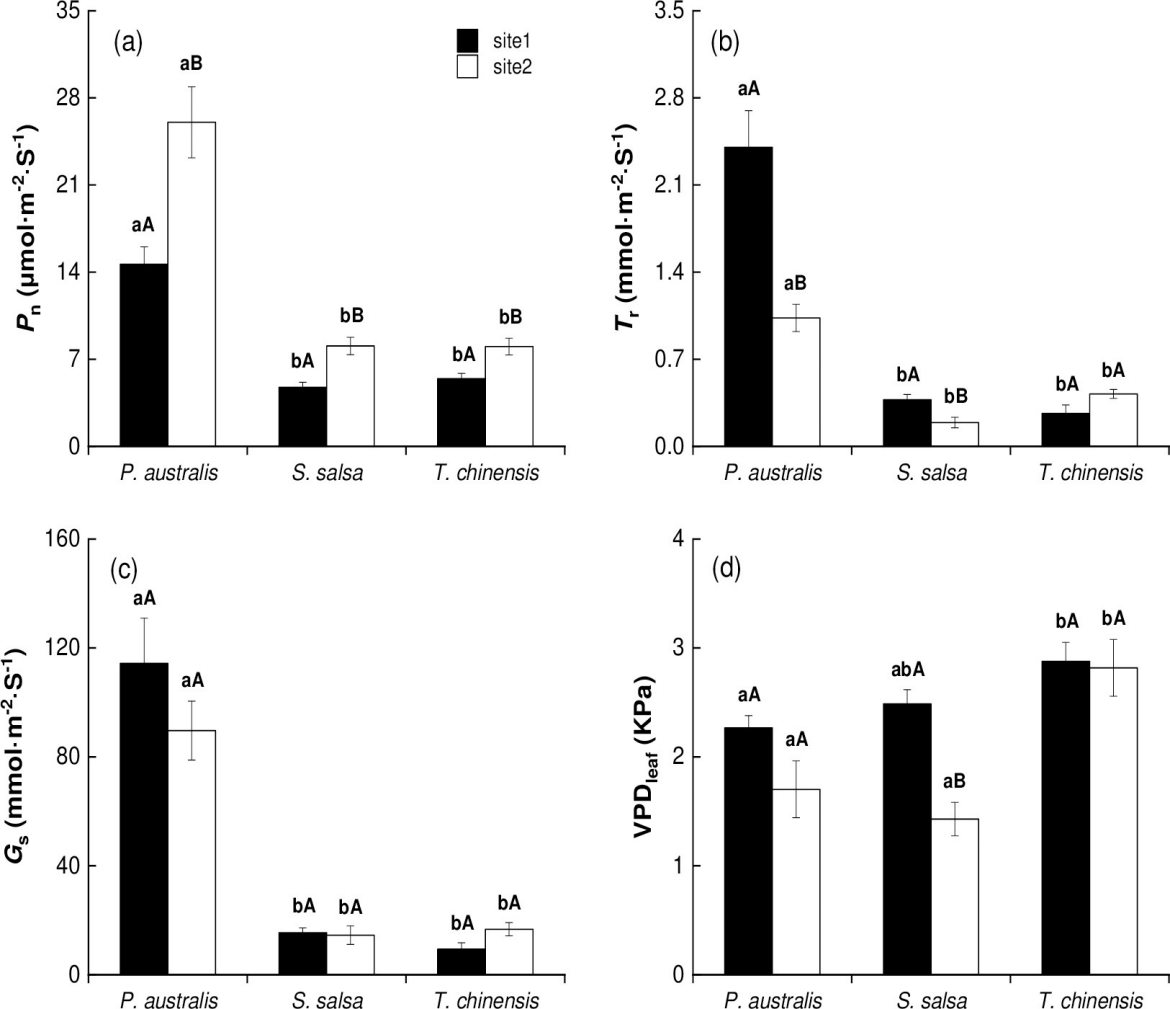
Gas exchange characters (mean ± SE) of halophytic plant communities. (a) Net photosynthetic rate (*P*_n_), (b) transpiration rate (*T*_r_), (c) stomatal conductance (*G*_s_), and (d) leaf vapor pressure deficit (VPD_leaf_). The different lower letters indicate significant differences (*P*<0.05) between the different species in the same site; the different upper letters indicate significant differences (*P*<0.05) between the same species in different sites.

*P*. *australis* had higher *P*_n_, *T*_r_, and *G*_s_ than *S*. *salsa* and *T*. *chinensis* (Fig 1A, 1B and 1C; *P* < 0.05). However, the differences between *S*. *salsa* and *T*. *chinensis* were not significant (*P* > 0.05). *T*. *chinensis* had higher VPD_leaf_ than *P*. *australis* and *S*. *salsa* in site 2 (Fig 1D, *P* < 0.05), but the differences between *P*. *australis* and *S*. *salsa* were not significant (Fig 1D, *P* > 0.05).

For identical species in different sites, *P*. *australis* in site 1 had lower *P*_n_ but higher *T*_r_ than *P*. *australis* in site 2 (Fig 1A and 1B, *P* < 0.05). *S*. *salsa* and *T*. *chinensis* in site 1 had lower *P*_n_ than those in site 2. However, the *T*_r_ and VPD_leaf_ of *S*. *salsa* were higher in site1 than in site 2 (Fig 1A, 1B and 1D; *P* < 0.05). The *G*_s_ of the three species had no significant differences between the two sites (Fig 1C, *P* > 0.05).

### LMA and leaf nutrient conditions of halophytes

**Fig 2:**
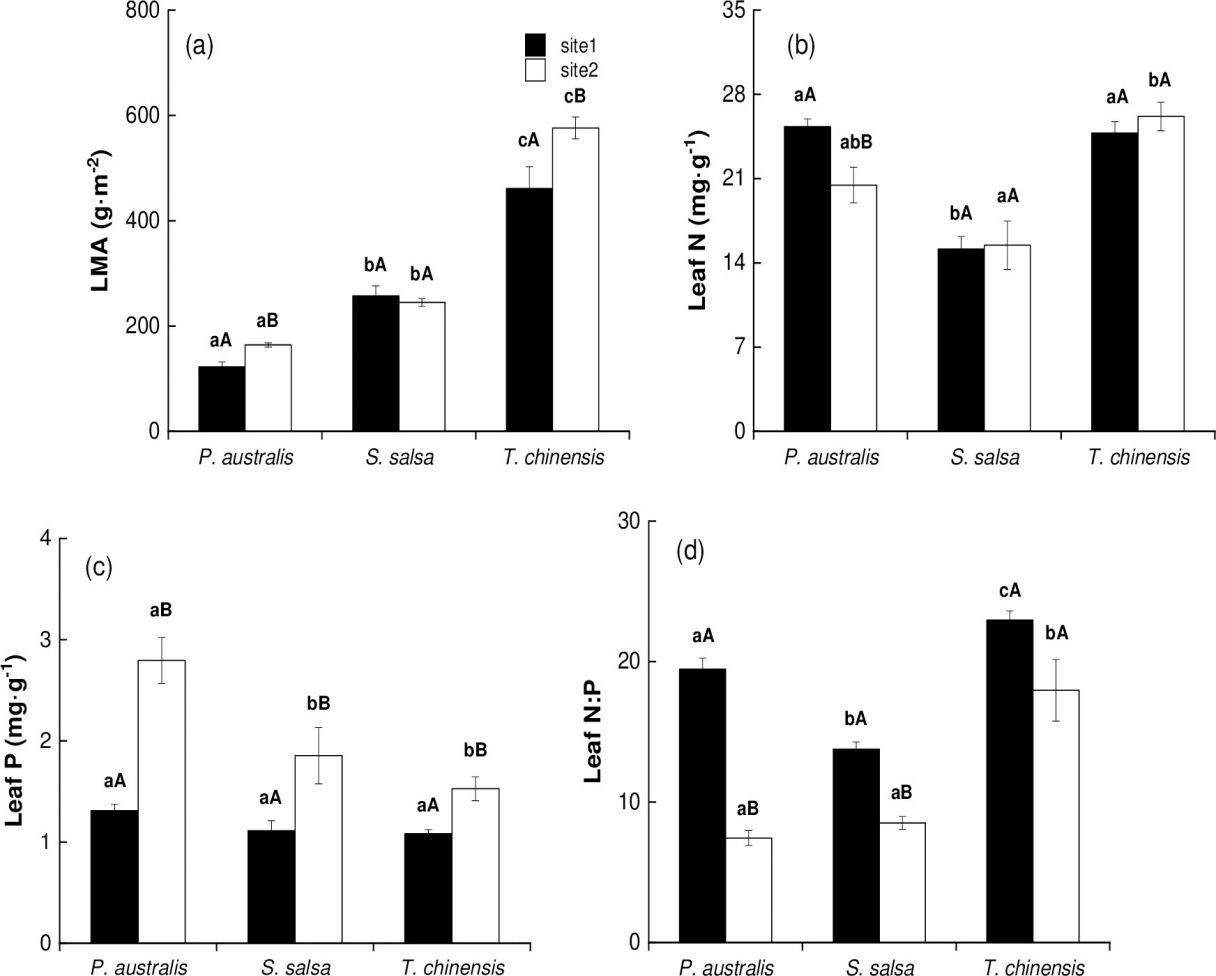
Leaf mass per area (LMA) and leaf nutrient conditions (mean ± SE) of halophytic plant communities. (a) LMA, (b) leaf N, (c) leaf P, and (d) leaf N:P. The different lower letters indicate significant differences (*P*<0.05) between the different species in the same site; the different upper letters indicate significant differences (*P*<0.05) between the same species in different sites.

For different species in site 1, the LMA was highest for *T*. *chinensis*, followed by *S*. *salsa* and *P*. *australis* (Fig 2A, *P* < 0.05). Leaf N was lowest for *S*. *salsa* but did not differ between *P*. *australis* and *T*. *chinensis* (Fig 2B, *P* > 0.05). The difference for leaf P among the three halophytic plant communities in site 1 was not significant (Fig 2C, *P* > 0.05). However, leaf N:P was highest for *T*. *chinensis*, followed by *P*. *australis* and then *S*. *salsa* (Fig 2D, *P* < 0.05).

For different species in site 2, the LMA was highest for *T*. *chinensis*, followed by *S*. *salsa* and *P*. *australis* (Fig 2A, *P* < 0.05). *T*. *chinensis* had higher leaf N than *S*. *salsa* (Fig 2B, *P* < 0.05), but the differences between *P*. *australis* and the two other communities were not significant (*P* > 0.05). Leaf P was highest for *S*. *salsa*, followed by *P*. *australis* and *T*. *chinensis* (Fig 2C, *P* < 0.05). Leaf N:P was highest for *T*. *chinensis* (Fig 2D, *P* < 0.05), followed by *S*. *salsa* and *P*. *australis*, but the difference between these two species was not significant (*P* > 0.05).

For identical species in different sites, *P*. *australis* in site 1 had lower LMA and leaf P, but higher leaf N and N:P than in site 2. *S*. *salsa* in site 1 had lower leaf P and higher N:P than in site 2. However, the differences for LMA and leaf N between the two sites were not significant. *T*. *chinensis* in site 1 had lower LMA and leaf P than in site 2 (Fig 2A and 2C, *P* < 0.05). However, the differences for leaf N and leaf N:P between the two study sites were not significant (Fig 2B and 2D, *P* > 0.05).

### Relationships between LMA and gas exchange characteristics

**Fig 3:**
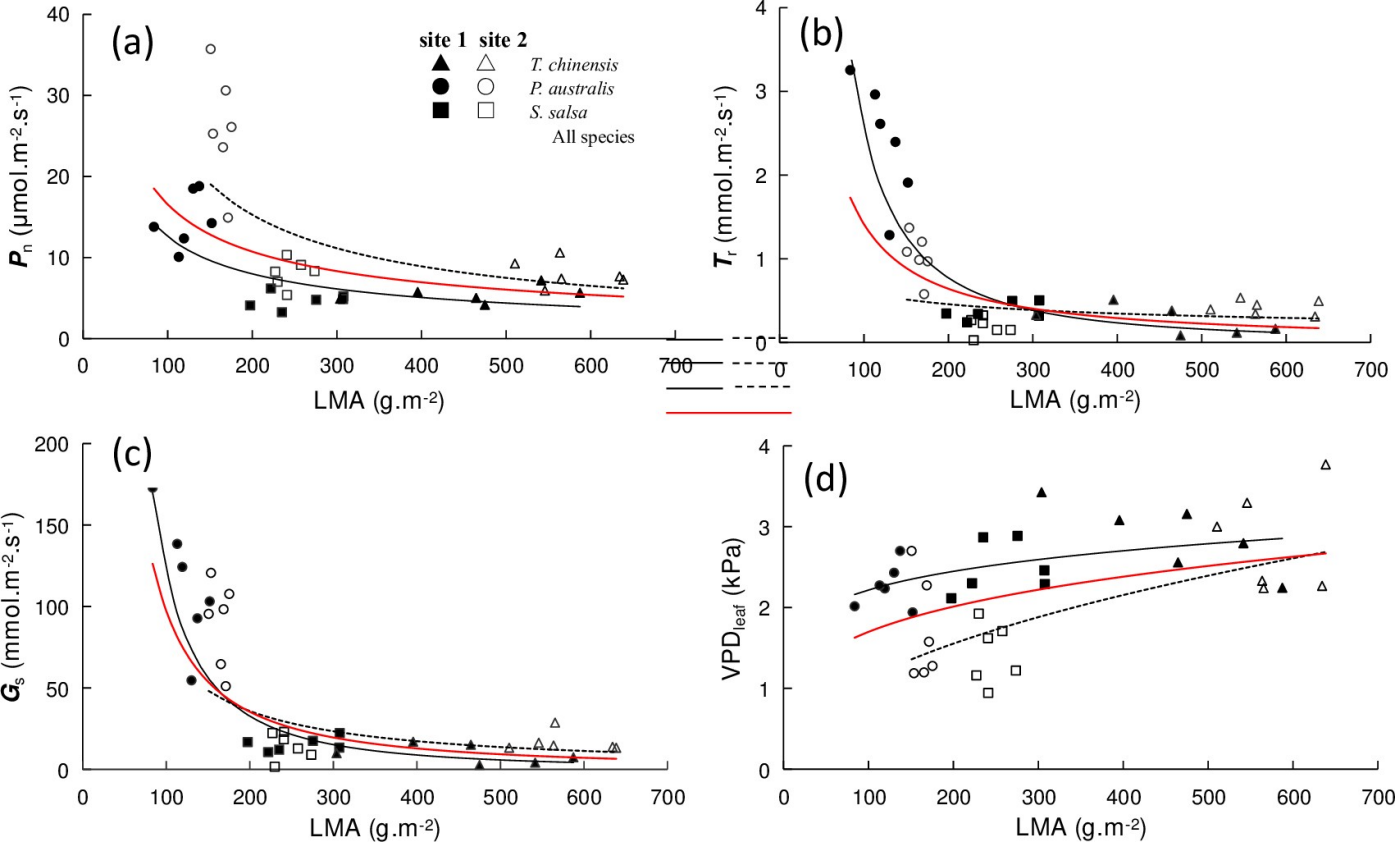
Relationships between LMA and gas exchange characteristics of halophytic plant communities. (a) LMA and *P*_n_ (equation for site 1: y = 255.59x^−0.654^, R^2^ = 0.489, *P* = 0.001; equation for site 2: y = 935.77x^−0.777^, R^2^ = 0.481, *P* = 0.001; equation for all species: y = 295.94x^−0.626^, R^2^ = 0.321, *P* < 0.001); (b) LMA and *T*_r_ (equation for site 1: y = 7037x^−1.721^, R^2^ = 0.792, *P* < 0.001; equation for site 2: y = 3.8727x^−0.403^, R^2^ = 0.053, *P* = 0.358; equation for all species: y = 268.35x^−1.139^, R^2^ = 0.375, *P* < 0.001); (c) LMA and *G*_s_ (equation for site 1: y = 729930x^−1.89^, R^2^ = 0.804, *P* < 0.001; equation for site 2: y = 9216.9x^−1.048^, R^2^ = 0.270, *P* = 0.027; equation for all species: y = 80893x^−1.46^, R^2^ = 0.511, *P* < 0.001); (d) LMA and VPD_leaf_ (equation for site 1: y = 1.1491x^0.1426^, R^2^ = 0.272, *P* = 0.026; equation for site 2: y = 0.1273x^0.4721^, R^2^ = 0.397, *P* = 0.005; equation for all species: y = 0.5527x^0.2437^, R^2^ = 0.160, *P* = 0.015).

Leaf *P*_n_ showed a significant decrease in power law with increasing LMA for species in site 1 (Fig 3A, *P* = 0.001, R^2^ = 0.489) and site 2 (Fig 3A, *P* = 0.001, R^2^ = 0.481), respectively. A similar trend was found for all species pooled together (Fig 3A, *P* < 0.001, R^2^ = 0.321). *T*_r_ showed a significant decrease in power law with increasing LMA for the species in site 1 (Fig 3B, *P* < 0.001, R^2^ = 0.792) and for all the species pooled together (Fig 3B, *P* < 0.001, R^2^ = 0.375). However, the changes for *T*_r_ with increasing LMA for species in site 2 were not significant (Fig 3B, *P =* 0.358, R^2^ = 0.053).

*G*_s_ showed a significant decrease in power law with increasing LMA for the species in sites 1 (Fig 3C, *P* < 0.001, R^2^ = 0.804) and 2 (Fig 3C, *P* = 0.027, R^2^ = 0.270) for all the species pooled together (Fig 3C, *P* < 0.001, R^2^ = 0.511). VPD_leaf_ showed a significant increase in power law with increasing LMA for the species in sites 1 (Fig 3D, *P* = 0.026, R^2^ = 0.272) and 2 (Fig 3D, *P* = 0.005, R^2^ = 0.397). A similar trend was found for all the species pooled together (Fig 3D, *P* = 0.015, R^2^ = 0.160).

### Relationships between leaf nutrient conditions and *P*_n_

**Fig 4:**
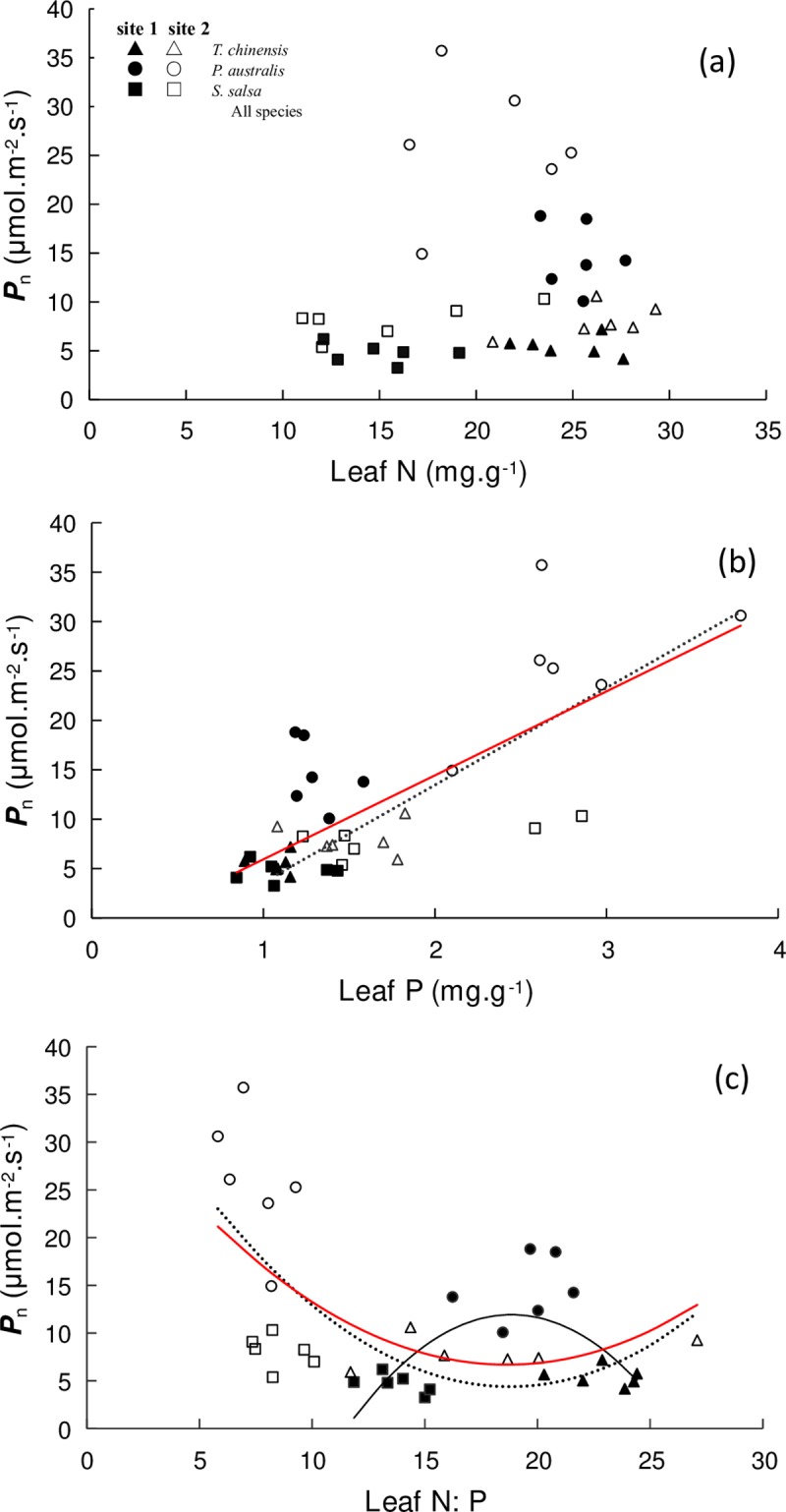
Relationships between leaf nutrient conditions and *P*_n_ of halophytic plant communities. (a) Leaf N and *P*_n_ (equations for sites 1 and 2 and all species: *P* > 0.05). (b) Leaf P and *P*_n_ (equation for site 1: *P* = 0.081; equation for site 2: y = 9.8482x − 6.2357, R^2^ = 0.589, *P* < 0.001; equation for all species: y = 8.5006x − 2.5626, R^2^ = 0.542, *P* < 0.001). (c) Leaf N:P and *P*_n_ (equation for site 1: y = −0.2213x^2^ + 8.3418x − 66.676, R^2^ = 0.377, *P* = 0.029; equation for site 2: y = 0.1112x^2^ − 4.1707x + 43.505, R^2^ = 0.372, *P* = 0.031; equation for all species: y = 0.0879x^2^ − 3.277x + 37.24, R^2^ = 0.306, *P* = 0.002).

The correlations found between leaf N and *P*_n_ were not significant (Fig 4A, *P* > 0.05). However, a significant linear relationship was found between Leaf P and *P*_n_ for the species in site 2 (Fig 4B, *P* < 0.001, R^2^ = 0.589). A similar trend was found for all the species pooled together (Fig 4B, *P* < 0.001, R^2^ = 0.542).

Leaf N:P showed a nonlinear relationship for the species in sites 1 (Fig 4C, *P* = 0.029, R^2^ = 0.377) and 2 (Fig 4C, *P* = 0.031, R^2^ = 0.372). For the species in site 2, *P*_n_ decreased until N:P increased to approximately 20 mg‧g^−1^ and then increased along with leaf N:P. However, the opposite trend was found for the species in site 1. For all species pooled together, the quadratic regression also suggested that *P*_n_ decreased until N:P increased to approximately 20 mg‧g^−1^ and then increased along with leaf N:P (Fig 4C, *P* = 0.002, R^2^ = 0.305).

### PNUE and PPUE of halophytes

**Fig 5:**
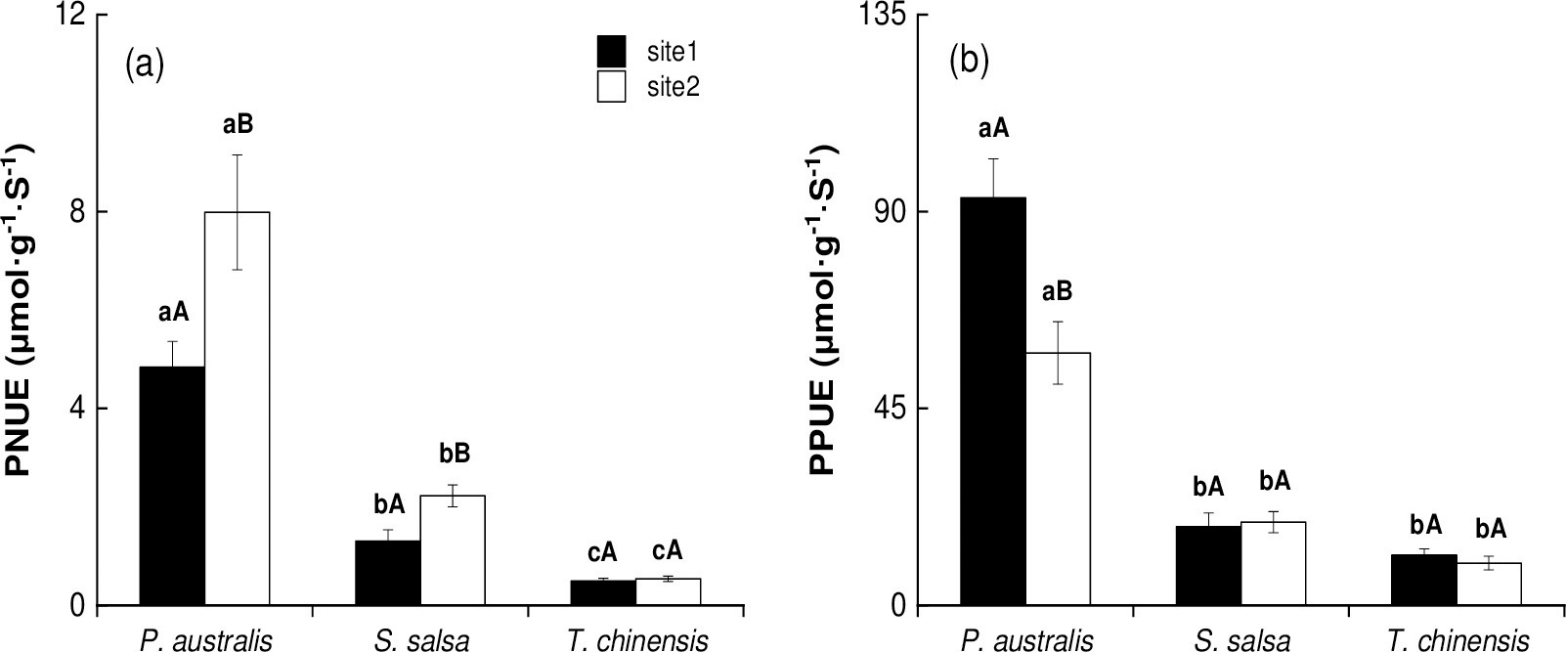
Photosynthetic N and P use efficiency (PNUE and PPUE; mean ± SE) of halophytic plant communities. (a) PNUE; (b) PPUE. The different lower letters indicate significant differences (*P*<0.05) between the different species in the same site; the different upper letters indicate significant differences (*P*<0.05) between the same species in different sites.

For different species at the same site, PNUE and PPUE were highest for *P*. *australis*, followed by *S*. *salsa* and *T*. *chinensis* (Fig 5A and 5B, *P* < 0.05). For identical species in different sites, *P*. *australis* in site 1 had lower PNUE but higher PPUE than site 2 (Fig 5A and 5B, *P* < 0.05). For *S*. *salsa*, PNUE was lower in site 1 than in site 2 (Fig 5A, *P* < 0.05). However, the differences for PPUE between the two study sites were not significant (Fig 5B, *P* > 0.05). For *T*. *chinensis*, PNUE and PPUE had no significant differences between the two sites (Fig 5A and 5B, *P* > 0.05).

### Relationships between photosynthetic nutrient use efficiency and *P*_n_

**Fig 6:**
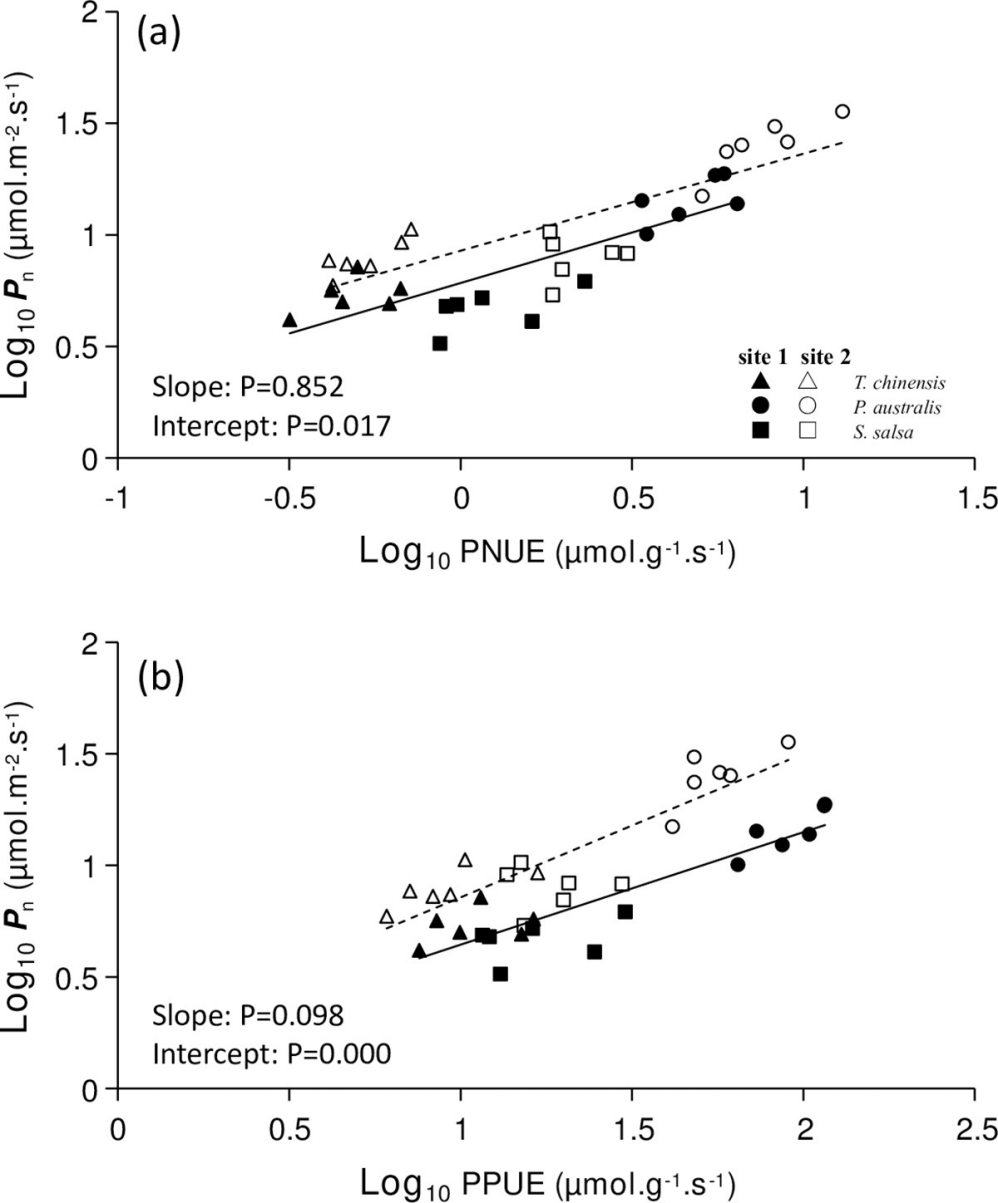
Relationships between photosynthetic nutrient use efficiency and *P*_n_ of halophytic plant communities. (a) PNUE and *P*_n_ (equation for site 1: y = 0.4522x+0.7842, R^2^ = 0.679, *P* < 0.001; equation for site 2: y = 0.4345x + 0.9289, R^2^ = 0.676, *P* < 0.001; with data log_10_-transformed); (b) PPUE and *P*_n_ (equation for site 1: y = 0.5042x + 0.1405, R^2^ = 0.812, *P* < 0.001; equation for site 2: y = 0.6439x + 0.2127, R^2^ = 0.752, *P* < 0.001; with data log_10_-transformed).

PNUE was positively correlated with *P*_n_ in sites 1 (Fig 6A, *P* < 0.001, R^2^ = 0.838) and 2 (Fig 6A, *P* < 0.001, R^2^ = 0.904). The slopes of PNUE-*P*_n_ did not significantly differ between the two regression lines (*P* > 0.05), with a common slope of 0.539 (95% CI = [0.439, 0.661]). However, the y-intercept of PNUE-*P*_n_ in site 2 was higher than that in site1 (*P* = 0.017). PPUE was positively correlated with *P*_n_ in sites 1 (Fig 6B, *P* < 0.001, R^2^ = 0.925) and 2 (Fig 6B, *P* < 0.001, R^2^ = 0.870). The slopes of PPUE-*P*_n_ did not differ between the two regression lines (*P* > 0.05), and the average was 0.631 (95% CI = [0.526, 0.762]). The y-intercept of PPUE-*P*_n_ in site 2 was also higher than that in site 1 (*P* < 0.001).

### Relationships between LMA and photosynthetic nutrient use efficiency

**Fig 7:**
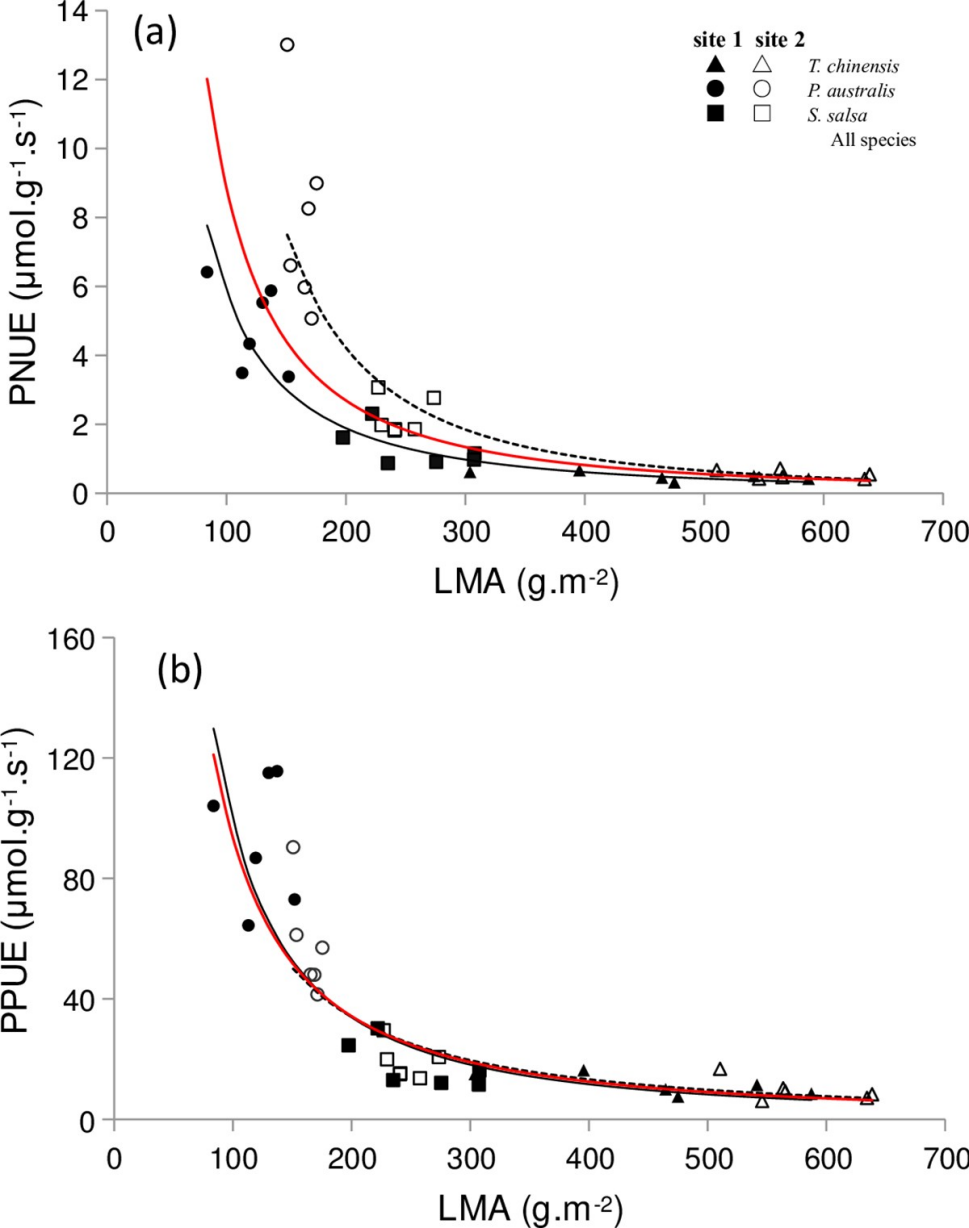
Relationships between LMA and photosynthetic nutrient use efficiency of halophytic plant communities. (a) LMA and PNUE (equation for site 1: y = 10339x^−1.625^, R^2^ = 0.909, *P* < 0.001; equation for site 2: y = 204171x^−2.036^, R^2^ = 0.923, *P* < 0.001; equation for all species: y = 24214x^−1.719^, R^2^ = 0.805, *P* < 0.001); (b) LMA and PPUE (equation for site 1: y = 118828x^−1.54^, R^2^ = 0.849, *P* < 0.001; equation for site 2: y = 45843x^−1.36^, R^2^ = 0.812, *P* < 0.001; equation for all species: y = 74475x^−1.451^, R^2^ = 0.832, *P* < 0.001).

PNUE showed a significant increase in power law when LMA decreased for the species in sites 1 (Fig 7A, *P* < 0.001, R^2^ = 0.908) and 2 (Fig 7A, *P* < 0.001, R^2^ = 0.923) and for all the species pooled together (Fig 7A, *P* < 0.001, R^2^ = 0.805). PPUE showed a significant increase in power law when LMA decreased for species in sites 1 (Fig 7B, *P* < 0.001, R^2^ = 0.849) and 2 (Fig 7B, *P* < 0.001, R^2^ = 0.812) and for all species pooled together in the two study sites (Fig 7B, *P* < 0.001, R^2^ = 0.832).

### Soil physicochemical properties in different sites

For different communities in site 1, *T*. *chinensis* had higher soil N and SWC but lower pH than *P*. *australis* and *S*. *salsa* (Table 1, *P* < 0.05). Soil salinity was higher in the community of *S*. *salsa* than in the communities of *P*. *australis* and *T*. *chinensis* (Table 1, *P* < 0.05). For soil P, the differences among the three communities were not significant (Table 1, *P* > 0.05).

**Table 1:** Soil physicochemical properties of halophytic plant communities in two sites.

Study sites	Community species	Soil N (mg.kg^-1^)	Soil P (mg.kg^-1^)	Salinity (‰)	SWC (%)	pH
Site 1	*P*. *australis*	0.63±0.06**aA**	0.52±0.07**aA**	9.95±2.02**aA**	37.02±1.13**aA**	8.47±0.09**aA**
	*S*.*salsa*	0.71±0.14**aA**	0.56±0.04**aA**	17.40±3.97**bA**	36.82±0.56**aA**	8.39±0.23**aA**
	*T*. *chinensis*	0.93±0.24**bA**	0.50±0.07**aA**	11.70±2.49**aA**	40.95±2.63**bA**	8.12±0.11**bA**
Site 2	*P*. *australis*	0.45±0.05**aB**	0.56±0.03**aA**	23.12±2.07**aB**	30.57±5.11**aB**	8.14±0.09**aB**
	*S*.*salsa*	0.59±0.10**bA**	0.61±0.04**bA**	27.40±5.28**abB**	24.68±8.57**aB**	8.21±0.08**aA**
	*T*. *chinensis*	0.59±0.09**bB**	0.53±0.03**aA**	29.05±5.23**bB**	26.97±4.36**aB**	8.26±0.18**aB**

The different lower letters indicate significant differences (*P*<0.05) between the different species in the same site; the different upper letters indicate significant differences (*P*<0.05) between the same species in different sites.

For different communities in site 2, *P*. *australis* had lower soil N and salinity than *S*. *salsa* and *T*. *chinensis* (Table 1, *P* < 0.05). Soil P was highest in the community of *S*. *salsa*, followed by the communities of *P*. *australis* and *T*. *chinensis*, which did not differ from each other. The differences for SWC and pH among the three communities were not significant (Table 1, *P* > 0.05).

For identical community in different sites, *P*. *australis* in site 1 had higher soil N, SWC, and pH but lower salinity than those in site 2 (Table 1, *P* < 0.05). Soil P did not differ between the *P*. *australis* communities in sites 1 and 2 (*P* > 0.05). For *S*. *salsa* communities, soil N, soil P and pH were common in the two sites. However, that community in site 1 had lower salinity and higher SWC than that in site 2 (Table 1, *P* < 0.05). *T*. *chinensis* had higher soil N and SWC but lower salinity and pH in site 1 than in site 2 (Table 1, *P* < 0.05), but the difference for soil P between the two sites was not significant (*P* > 0.05).

### Effects of site, species, and their interaction on leaf traits

The results of two-way ANOVA with the effects of site, species, and their interactions on leaf functional traits showed that site had no effect on *G*_s_ and leaf N but had a significant effect on other functional traits (Table 2, *P* < 0.001). Species had a significant effect on all leaf photosynthetic functional traits (Table 2, *P* < 0.001). Except for leaf P and leaf N:P, all *F*-ratios for species were larger than those for site, thereby indicating a high explanation for species. In this analysis, all leaf functional traits (except for *G*_s_) were also influenced by the interactions between site and species. However, the *F*-ratios for site × species were lower than those for site and species separately, thereby suggesting a slightly weak explanation for their interactions (Table 2, *P* < 0.01).

**Table 2:** Results of a two-way analysis of variance to test the effects of site, species, and their interactions on the leaf traits.

Leaf traits	Site	Species	Site × Species
*P*_n_ (μmol.m^-2^.s^-1^)	26.263**	66.477**	6.304**
*T*_r_ (mmol.m^-2^.s^-1^)	18.026**	73.154**	17.952**
*G*_s_ (mmol.m^-2^.s^-1^)	0.803NS	73.996**	1.982NS
VPD_leaf_ (kPa)	12.837**	13.937**	3.398*
LMA (g.m^-2^)	7.798**	168.395**	4.617*
Leaf N (mg.g^-1^)	0.999NS	33.467**	3.334*
Leaf P (mg.g^-1^)	44.882**	11.501**	5.424*
Leaf N:P ratio	75.192**	42.808**	7.213**
PNUE (μmol.g^-1^.s^-1^)	9.700**	66.457**	4.409*
PPUE (μmol.g^-1^.s^-1^)	8.727**	99.950**	8.232**

*F*-ratios are shown in the table

**indicates significant at 0.01 level

* indicates significant at 0.05 level, NS indicates no significant effect

### RDA for gas exchange characters and their influencing factors

**Fig 8:**
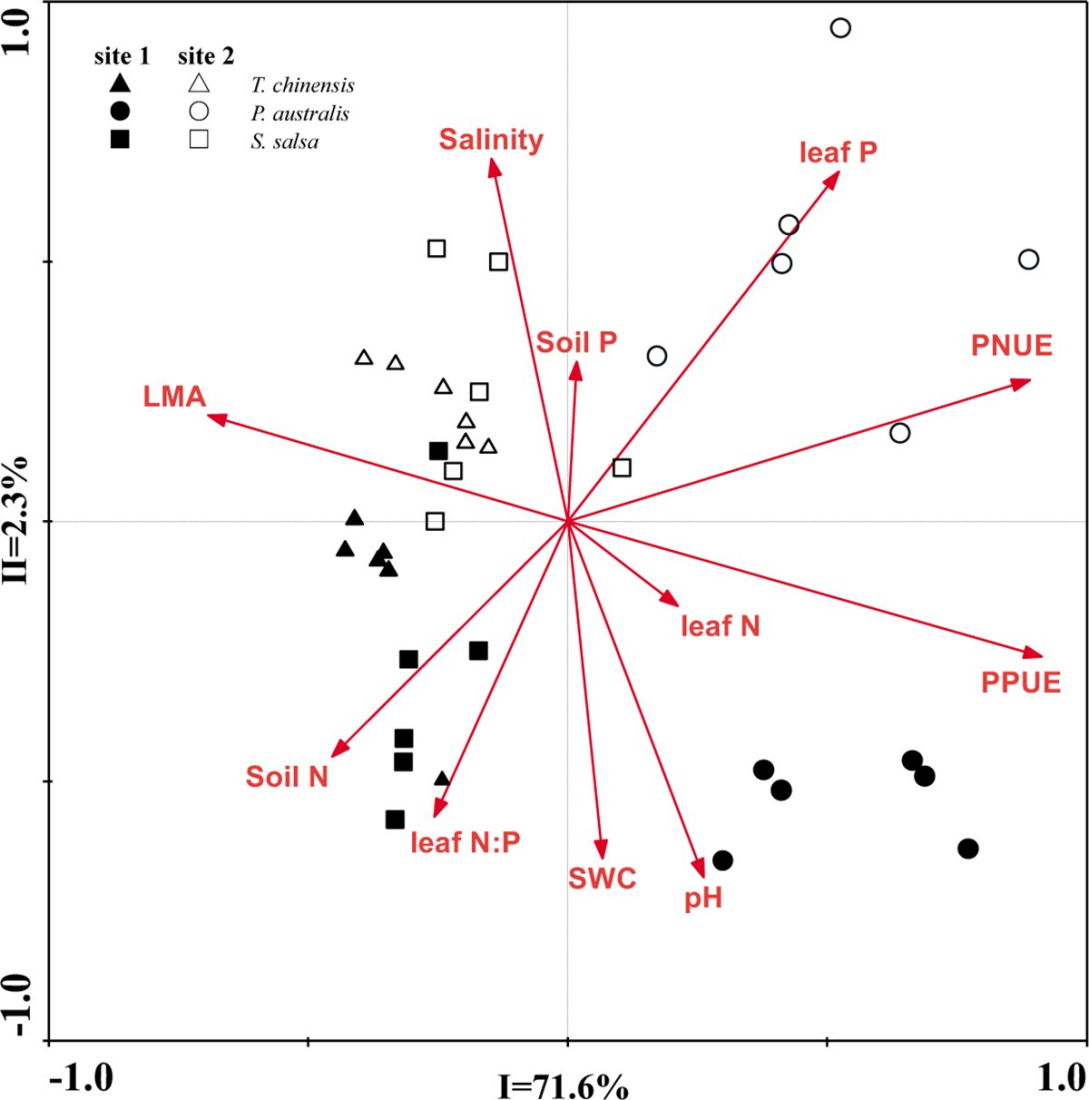
Redundancy analysis (RDA) of relationships between gas exchange characteristics and the influencing factors of halophytic plant communities.

The RDA results showed that the explanatory of the four gas exchange characteristics for all halophytic plant communities in two sites were 71.6% and 2.3% (Fig 8), that is, the two axes explained 73.9% variations of the gas exchange capacity. Most of the information on the gas exchange characteristics for halophytic plant communities and their influencing factors could be reflected by the first two axes.

The Monte-Carlo test results showed that the important values (*F*) of the influencing factors decreased in the order of PPUE > PNUE > LMA > leaf P > soil N > pH > leaf N:P > leaf N > salinity > SWC > soil P (Table 3). The effects for PPUE (Table 3, *P* = 0.002), PNUE (*P* = 0.002), LMA (*P* = 0.002), leaf P (*P* = 0.002), and soil N (*P* = 0.02) were significant, and their explanations were 60.0%, 57.1%, 35.0%, 22.0%, and 15.8%, respectively (Table 3). In contrast with these factors, the effects for pH, leaf N:P, leaf N, salinity, SWC, and soil were not significant P (Table 3, *P* > 0.05). PPUE, PNUE, LMA, leaf P, and soil N were the main driving factors of gas exchange capacity for all communities in the two sites.

**Table 3:** Importance ranking and significance of influencing factors in their explanation on the gas exchange capacity of halophytic plant communities.

Influencing factors	Importance ranking	Explanation (%)	*F*	*P*
PPUE	1	60.0	51.095	**0.002**
PNUE	2	57.1	45.222	**0.002**
LMA	3	35.0	18.295	**0.002**
Leaf P	4	22.0	9.383	**0.002**
Soil N	5	15.8	6.395	**0.02**
pH	6	7.1	2.587	0.08
Leaf N:P	7	6.9	2.527	0.082
Leaf N	8	4.1	1.471	0.23
Salinity	9	3.9	1.378	0.24
SWC	10	2.4	0.824	0.338
Soil P	11	0.7	0.247	0.732

Significant results are shown in bold (*P* < 0.05).

### N:P thresholds for halophytes in different sites

**Fig 9:**
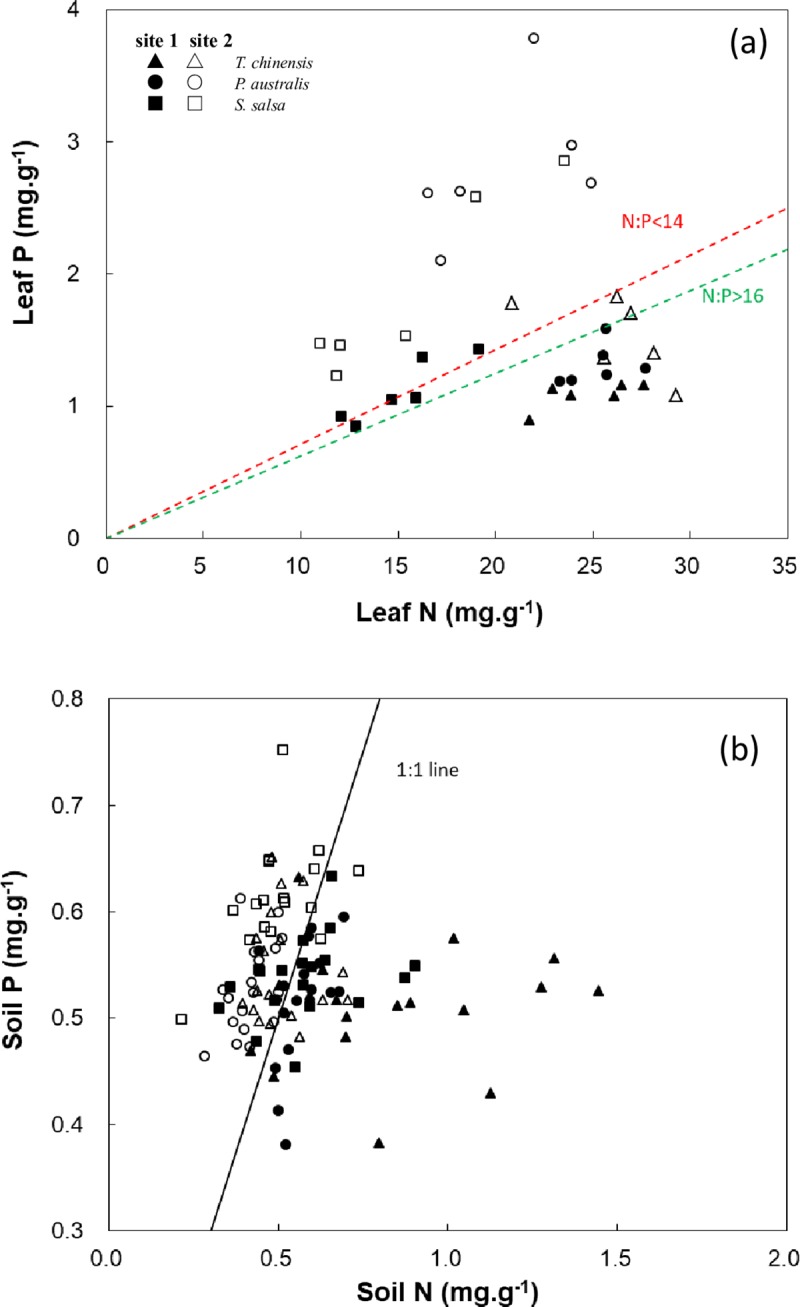
Relationships between N and P of plants and soils. (a) N versus P of plants; (b) N versus P of soils.

For the species in site 1, the N:P thresholds of *P*. *australis* and *T*. *chinensis* were higher than 16 (Fig 9A), which indicated the P limitation for the two species in this habitat. However, all the N:P thresholds of *S*. *salsa* were lower than 16, and 50% of the thresholds were lower than 14 (Fig 9A), thereby suggesting N limitation or ambiguous limitation for *S*. *salsa* in site 1.

For species in site 2, The N:P thresholds of *P*. *australis* and *S*. *salsa* were lower than 14 (Fig 9A), which indicated a N limitation for the two species in this habitat. For *T*. *chinensis* in site 2, 50% of the thresholds were higher than 16 (Fig 9A), thereby suggesting that *T*. *chinensis* was grown under P-limited conditions in site 2. However, a sample with a N:P of <16 can either show N limitation or ambiguous limitation for *T*. *chinensis* in site 2.

For soil samples in the communities of site 1, 75.93% of the N:P thresholds were higher than 1 (Fig 9B), which indicated that a large N input occurred in these habitats. However, for all the soil samples in the communities of site 2, only 12.96% of the N:P thresholds were higher than 1 (Fig 9B), which suggested that the soil was rich in P in these habitats.

## Discussion

### Gas exchange capacity and leaf nutrient conditions

Photosynthesis is an important indicator for the estimation of plant productivity. However, the photosynthetic rate is not enough to elucidate the gas exchange capacity of a plant. In many studies, the leaf functional traits (i.e., *P*_n_, *T*_r_, *G*_s_ and VPD_leaf_) and leaf structural traits (i.e., LMA, leaf N and P) are generally used to evaluate the carbon assimilation patterns of different ecosystems [39–41]. The current study’s results indicated that *P*. *australis* had higher *P*_n_, *T*_r_ and *G*_s_ than *S*. *salsa* and *T*. *chinensis* (Fig 1) in both sites, which may have been affected by the genera and the environmental conditions. However, the gas exchange characteristics are intrinsic, and environmental determinants remain unclear in previous studies [4]. No direct evidence showed positive connections between soil properties and photosynthetic rate in each site. For example, *P*. *australis* had lower soil N and SWC but higher *P*_n_, *T*_r_, and *G*_s_ than *T*. *chinensis* in site 1 (Table 1; Fig 1). This result indicated that those redundant nutrients and water are generally used to synthesize compounds for stress resistance in the saline–alkaline habitat. For different sites, the communities in site 1 had higher soil N and SWC and lower salinity than those in site 2 (Table 1). However, *P*_n_ was lower in site 1 than in site 2 (Fig 1). The photosynthetic capacity was determined by the surroundings and influenced by genetic factors. To some extent, the photosynthetic capacity and water use efficiency of the plants are determined by their intrinsic factors under specific environmental conditions [42]. In this research, the *F*-ratios of species are higher than the site, and their interaction also confirmed the inference in Table 2.

Leaf N and P are two important intrinsic factors related to photosynthesis. These factors are relatively constant in the given environmental conditions and reflect highly adaptable strategies for plants in carbon fixation [17, 41]. Therefore, exploring the relationships between gas exchange characters and leaf traits are crucial in understanding the morphogenesis and evolution of halophytes. The photosynthetic rate of plants has significant positive correlations with N and P concentrations in leaves [6–10]. In the present study, *P*_n_ did not have a significant correlation with leaf N in both sites, which indicated that the *P*_n_ might not have changed with increasing soil N. However, the Monte-Carlo test in RDA showed that soil N had a significant effect on the gas exchange capacity of halophytes (Table 3, *P* = 0.02). This result may be correlated with N absorption and utilization strategies. The photosynthetic rate of two herbaceous did not significantly differ upon the application of nitrogen fertilizer [16]. The electron transport of chloroplast thylakoid membrane and the other sections involved in the photoreaction is also unaffected by nitrogen nutrition [43]. By contrast, leaf P had a positive correlation with *P*_n_ in site 2. However, that relationship was weak in site 1 (Fig 4B). The differences in leaf P-*P*_n_ between the two sites may be related to their different nutrient limitation conditions. The difference for soil P between the two sites was not significant, but soil N was significantly higher in site 1 than in site 2 (Table 1), which indicated a high N:P ratio in the soil of site 1 (Fig 9). As suggested in previous studies [44], the high input ratios of N and P result in P limitation in site 1. Meanwhile, P is easily decoupled from C and N cycles under P-limited conditions [32, 45]; this finding can explain the phenomena in our research.

### Gas exchange capacity and LMA

The photosynthetic rate is tightly connected with LMA in many terrestrial ecosystems [6, 10, 40]. In our research, the gas exchange characteristics of the two study sites showed no evident changes with leaf nutrient conditions. However, the two study sites were significantly affected by LMA in an exponential manner (Fig 3A, 3B, 3C and 3D). In most situations, wetland plants are generally expressed through physiological, morphological, and genetic changes in response to waterlogging stress [46, 47]. All species tend to maintain their gas exchange characteristics by optimizing LMA [40], which might be a possible strategy for halophytes to adapt to an environment with short-term flooding. In the present study, LMA was highest for *T*. *chinensis* (461.31 and 576.34 g‧m^−2^ in sites 1 and 2, respectively), followed by *S*. *salsa* (257.49 and 244.97 g‧m^−2^, respectively) and *P*. *australis* (122.56 and 164.15 g‧m^−2^, respectively). As a woody plant, the LMA of *T*. *chinensis* is two- to threefold higher than that of annual and perennial herbs (*S*. *salsa* and *P*. *australis*), which proves that the LMA of trees and shrubs is larger than herbs [10]. In summary, the morphological structure of the plant leaves in coastal wetlands differs from those in other ecosystems. The leaves of halophytes are generally small in size, mostly narrow or coniferous, scaly, and fleshy due to flooding and salinity stress [47]. These characteristics result in considerable changes in LMA. The large variation of LMA among halophytes may be the main reason of the anomalous fluctuations in their gas exchange characteristics. RDA result supported our deduction and indicated that LMA has negative and significant effect on the gas exchange capacity of halophytes. By definition, the larger the LMA is, the higher proportion of the biomass allocated to the leaves per unit area will be [40, 41]. Thus, high LMA has a dilution effect on the nutrient content of per leaf area, which reduces their gas exchange capacity of halophytes. Here, the explanation of LMA was 35% (Table 3), which was lower than those of PPUE and PNUE but higher than those of other influencing factors. In this scenario, LMA was tightly coupled with PNUE and PPUE in both sites (Fig 7), thereby suggesting that photosynthetic nutrient use efficiency determined the gas exchange capacity of halophytes.

### Gas exchange capacity and photosynthetic nutrient use efficiency

Photosynthesis is related to the nutrients in leaves and leaf structure and to the photosynthetic nutrient use efficiency [41, 48, 49]. The PNUE and PPUE of halophytic plant communities in the two sites showed the same trend as *P*_n_, that is, *P*. *australis > S*. *salsa > T*. *chinensis* (Fig 5A and 5B). In many studies, PPUE and PNUE proved to be the important indicators of plant response to soil nutrient status [48, 49]. For example, plants tend to enhance their nutrient use efficiency when soil nutrients are scarce. However, low nutrient use efficiency indicates that plant communities in this habitat are not restricted by such nutrients [50–52]. In the present study, in site 2, soil N was low, and the leaf N:P ratios of *P*. *australis* and *S*. *salsa* were smaller than 14 (Fig 9A), which indicated that those herbs were limited by N in this site. The high PNUE of *P*. *australis* and *S*. *salsa* in site 2 was an adaptive strategy for plant in N-limitation habitat. As previously discussed, the increased soil N and P inputs in site 1 may lead to a shift from N-limited to P-limited situations, which were often found in the estuarine and coastal wetlands. Here, the leaf N:P ratios of *P*. *australis* and *T*. *chinensis* in site 1 were larger than 16, which also provided additional evidence for this scenario.

The curves of PNUE-*P*_n_ and PPUE-*P*_n_ fitted significantly in sites 1 and 2 (Fig 6A and 6B), respectively, which indicated that the photosynthetic nutrient use efficiency was more useful than nutrient concentrations in affecting plant photosynthesis. The shift in the slope and intercept of the fitting curves between leaf traits represents certain biological significance [9, 49]. In the present study, the slopes of PNUE-*P*_n_ and PNUE-*P*_n_ were not significant different between the two sites (Fig 6A and 6B), which indicated that the nutrient utilization strategy of the two sites was indiscriminate. By contrast, the y-intercepts of PNUE-*P*_n_ and PNUE-*P*_n_ were significantly higher in site 2 than in site 1 (Fig 6A and 6B), which indicated that the plant in site 1 had higher PNUE and PPUE than those in site 2 at a given amount of carbon assimilation. Hence, the plants in site 1 tend to allocate minimal N and P to photosynthetic apparatus in keeping similar carbon assimilation. RDA result suggested that PPUE and PNUE were the first two influencing factors in depicting the gas exchange capacity of halophytes in both sites (Fig 8, Table 3). Thus, halophytes may promote their photosynthetic nutrient use efficiency and decrease the assimilated allocation per leaf area to cope with the nutrient limitation changes in coastal wetlands.

## Conclusions

The photosynthetic capacity of halophytes was not primarily controlled by nutrient concentration in leaves. It depended on photosynthetic nutrient use efficiency. PNUE and PPUE showed a significant decrease in power law with increasing LMA for all the species in both sites. Thus, halophytes may promote their photosynthetic nutrient use efficiency and decrease the assimilated allocation per leaf area to cope with the nutrient limitation changes in coastal wetlands. RDA showed that the gas exchange capacity of the halophytic plant communities was significantly affected by PPUE (60.0% of explanation), PNUE (57.1%), LMA (35.0%), leaf P (22.0%), and soil N (15.8%). The plant productivity of the terrestrial plants depended more on the availability of nutrients than their concentrations under climate change conditions, thereby helping elucidate the leaf economic spectrum and physiological principles of halophytes in coastal wetlands.

## Supporting information

S1 Dataset This is the data of the current manuscript. (XLSX)
